# Frailty as a Predictor of Acute Kidney Injury in Hospitalized Elderly Patients: A Single Center, Retrospective Cohort Study

**DOI:** 10.1371/journal.pone.0156444

**Published:** 2016-06-03

**Authors:** Seon Ha Baek, Sung Woo Lee, Sun-wook Kim, Shin young Ahn, Mi-Yeon Yu, Kwang-il Kim, Ho Jun Chin, Ki Young Na, Dong-Wan Chae, Sejoong Kim

**Affiliations:** Department of Internal Medicine, Seoul National University Bundang Hospital, Seongnam, Republic of Korea; National Cancer Institute, UNITED STATES

## Abstract

**Background:**

Elderly patients have an increased risk for acute kidney injury (AKI). However, few studies have reported on predictors for AKI in geriatric patients. Therefore, we aimed at determining the effect of frailty as a predictor of AKI.

**Methods:**

We retrospectively enrolled 533 hospitalized elderly patients (aged ≥ 65 years) who had their creatinine levels measured (≥ 1 measurement) during admission for a period of 1 year (2013) and conducted a comprehensive geriatric assessment (CGA) within 1 year before the index hospitalization. We examined five variables (activity of daily living [ADL] and instrumental ADL dependence, dementia, nutrition, and polypharmacy) from CGA. We categorized the patients into 3 groups according to the tertile of aggregate frailty scores: Group 1, score 1–2; Group 2, score 3–4; Group 3, score 5–8).

**Results:**

Fifty-four patients (10.1%) developed AKI (median duration, 4 days). The frailest group (Group 3) showed an increased risk of AKI as compared to Group 1, (hazard ratio [HR] = 3.536, *P* = 0.002). We found that discriminatory accuracy for AKI improved with the addition of the tertile of aggregate frailty score to covariates (area under the receiver operator characteristics curves [AUROC] 0.641, AUROC 0.739, P = 0.004). Forty-six patients (8.6%) were transferred to nursing facilities and 477 patients (89.5%) were discharged home. The overall 90-day and 1-year mortality for elderly inpatients were 7.9% and 26.3%. The frailest group also demonstrated an increased risk of discharge to nursing facilities, and 90-day and 1-year mortality as compared to Group 1, independent of AKI severity (nursing facilities: odd ratio = 4.843, *P* = 0.002; 90-day mortality: HR = 6.555, *P* = 0.002; 1-year mortality: HR = 3.249, *P* = 0.001).

**Conclusions:**

We found that frailty may independently predict the development of AKI and adverse outcomes in geriatric inpatients.

## Introduction

Acute kidney injury (AKI) is a challenging medical complication with a high risk of morbidity and mortality. The incidence of AKI and severe AKI (requiring dialysis) has been increasing over the past decade[1–3]. A significant factor contributing to this increase is the old age of population, which is considered an independent risk factor for AKI[4]. Furthermore, elderly patients with AKI have worse renal recovery rate and higher mortality rate than younger patients with AKI [5, 6]. However, few studies have reported on predictors for AKI in geriatric patients, although knowing this information contributes to an early diagnosis of AKI and reducing the related medical costs.

Elderly patients vary widely in their fitness status, ranging from fit to frail[7]. The concept of frailty has been recently introduced to predict the risk of adverse outcomes in geriatric population[7]. Frailty is a state of decreased physiological reserves and results in an increased risk of mortality and hospitalization in various conditions such as community-dwelling patients and surgical patients[7–11]. However, the effects of frailty on the development of AKI in hospitalized elderly patients are unknown.

The comprehensive geriatric assessment (CGA) is a systematic multidimensional assessment aimed at identifying a frail person’s somatic, functional, psychological, and social features to improve diagnostic accuracy and develop a therapeutic plan. Recently, its value has been demonstrated in geriatric medicine[11, 12].

In the current study, we used the CGA to investigate the role of frailty as an independent risk factor for AKI and prognostic factor for clinical outcomes in elderly inpatients.

## Methods

### Study population

**Fig 1:**
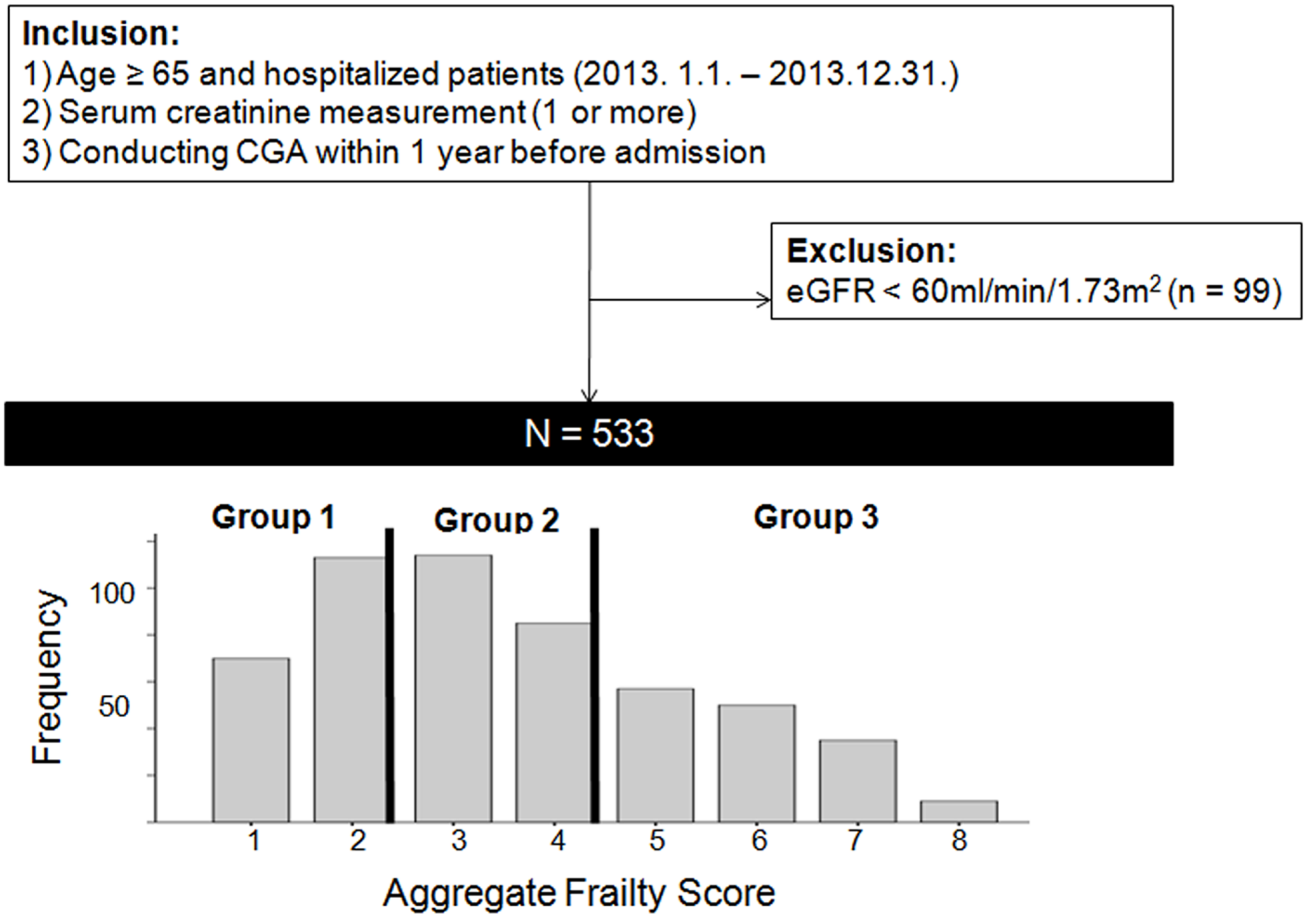
Study population and distribution of frailty score. CGA, comprehensive geriatric assessment; eGFR, estimated glomerular filtration rate. Group 1, 1–2; Group 2, 3–4; Group 3, 5–8.

We performed a single-center retrospective cohort study of 533 geriatric inpatients (aged ≥ 65 years) who had their creatinine (Cr) levels measured(≥ 1 measurement) during admission at the Seoul National University Bundang Hospital, a tertiary facility for a period of 1 year (January 1, 2013 through December 31, 2013). The patients who underwent CGA within 1 year before development of AKI were included. Patients were admitted owing to acute or chronic illness complications or surgery. We excluded those who were previously diagnosed with end-stage renal disease, or had a baseline value of estimated glomerular filtration rate (eGFR) < 60 ml/min/1.73 m^2^. We also excluded patients for whom complete CGA data were missing (Fig 1). If the patients included in the study were admitted to the hospital more than once, only the first admission was considered. All clinical investigations were conducted according to the 2008 Declaration of Helsinki and good clinical practice guidelines. This study was approved by the institutional review board of Seoul National University Bundang Hospital (IRB number: H-1508-310-115) with no written consent because patients records/information was anonymized and de-identified prior to analysis.

### CGA protocol

CGA performed within 1 year at least before the index hospitalization was considered to reflect usual biological status of the patients. The complete items of our CGA set have been described previously [11]. Our CGA protocol consisted of six domains: burden of comorbidity, polypharmacy, physical function, psychological status, nutrition, and risk of postoperative delirium. The functional status was assessed by activities of daily living (ADLs) and instrumental ADLs (IADLs). The ADLs were measured using modified Barthel Index, which includes 10 items (grooming, bathing, eating, dressing, toilet use, fecal and urinary continence, ability to climb up and down stairs, and walking in a hallway) [13]. This index ranges from 0 to 100, with 100 indicating full independence; 75–99, partial dependence; and < 75, full dependence. The IADLs were assessed using Lawton and Brody Index, which consists of 5 items for men (using telephone, shopping, travelling via car or public transportation, use of medication, and financial management). For women, three additional items (ability to prepare food, do laundry, and housekeeping) were assessed [14]. Patients with at least 1 dependent IADL were categorized as IADL dependent. Cognitive function was analyzed using Korean version of the Mini-Mental State Examination, with scores ranging from 0 to 30 (≥ 25, normal; 17–24, mild cognitive impairment; < 17, dementia)[15]. Depression was evaluated using the short form of Korean Geriatric Depression Scale, with scores ranging from 0 to 15 (≥ 10, severe depression and 5–9, mild depression)[16]. Nutritional status was examined using Mini Nutritional Assessment, with scores ranging from 0 to 30 (17–23.5, risk of malnutrition and < 17, malnutrition)[17]. Polypharmacy was defined as taking more than 5 drugs regularly, and inappropriate medication was determined by the Beers criteria[18]. The risk of delirium was evaluated by the Nursing Delirium Screening Scale with scores ranging from 0 to 5 (≥ 2, increased risk of postoperative delirium)[19].

### Measurements and definitions

Patients’ data were collected retrospectively via review of their electronic medical records. Serum Cr values were measured by the alkaline picrate Jaffe’s kinetic method using an automatic analyzer (Toshiba-200FR; Tokyo, Japan). The eGFR was calculated using the Modification of Diet in Renal Disease (MDRD) study equation[20] adjusted for Korean ethnic coefficient[21]. We assessed the comorbidities based on the code of the International Classification of Disease, 10th Revision *(ICD-10)* as follows: cardiovascular disease including angina pectoris (I20), myocardial infarction (I21–I23), ischemic heart disease (I24–I25), and heart failure (I50); cancer (C); cerebrovascular disease (I63–I66); hypertension (I10–I15); and diabetes mellitus (E10–14). Presence of hypertension and diabetes mellitus were also confirmed by using antihypertensive medication and antihyperglycemic agents, respectively. We retrospectively collected the main diagnosis at discharge as follows: cardiovasculocerebral disease included arrhythmia, heart failure, angina pectoris, myocardial infarction, ischemic heart disease, pulmonary thromboembolism, aortic aneurysm, and cerebral infarction; infection at any site; musculoskeletal disease including fracture at any site, degenerative arthritis, and herniated intervertebral disc; non-infection gastrointestinal disease including biliary stone without infection, inflammatory bowel disease, bleeding, ileus, and ischemic colitis; and neuropsychiatric disease including depression, somatiform disorder, alzheimer’s disease, and parkinsonism.

### Study Outcomes

#### AKI definition and severity

To detect AKI, at least 2 Cr measurements were required: one during the baseline time window and a second one on a different day during hospital admission. We defined the baseline Cr levels using 6-month Cr data from before patients’ admission, because this directly affects the detection and staging of AKI as follows: the lowest Cr value was that measured < 90 days prior to admission. If this value was not available, the lowest Cr value measured between 90 and 180 days before admission was considered. If this value was also unavailable, the Cr level was estimated using the MDRD study equation[20], adjusted for Korean ethnic coefficient[21], assuming that the baseline eGFR is 75ml/min/1.73m^2^[22]. Inpatient Cr measurements were defined as those obtained during admission. Using a baseline and an inpatient Cr value measured on different days, we categorized the patients into two groups: with AKI and without AKI. Per the serum Cr criteria proposed by Kidney Disease: Improving Global Outcomes (KDIGO)[22], a patient was assigned to the “with AKI” group if any inpatient Cr measurement was ≥ 26.52μmol/L greater than the baseline value or ≥ 1.5-fold higher than the baseline value. AKI severity was defined using KDIGO stages based on the baseline and maximum inpatient Cr value. We defined AKI solely using changes in measured serum Cr value because urine output data were not consistently available for all inpatients. We defined severe AKI as stage 2 and 3 AKI using the KDIGO stages.

#### Discharge to nursing facilities

We defined discharge to nursing facilities as discharge to a nursing home, transitional care, or any long-term care facility if the patient had lived at home before being admitted.

#### Ninety-day (short-term) and 1-year (long-term) all-cause mortality (ACM) rate

We combined mortality data from the Ministry of Security and Public Administration with our data set, by using each individual’s unique identifier. Mortality data were obtained until August 31, 2015.

### Statistical analysis

Continuous variables were expressed as mean ± standard deviation or as percentages for categorical variables. The differences in continuous variables were analyzed using Student’s *t*-tests and one-way analysis, while chi-square tests and Fisher’s exact test were used for categorical variables. To assess multicollinearity among explanatory variables used to make an aggregated frailty score, we used variance inflation factor. Cox’s hazard proportion analysis was used to estimate the hazard ratios (HRs) for AKI according to the tertile of frailty score. We conducted the test of proportional hazards assumptions and restricted cubic spline curves. A logistic regression analysis was used to evaluate the risk of discharge to nursing facility and ACM according to the tertile of frailty score. The HRs, odds ratios (ORs), and 95% confidence interval for the development of AKI, discharge to a nursing facility, and ACM were calculated after stepwise adjustment for multiple confounders. To estimate whether the discriminatory accuracy for outcomes improves with the addition of frailty score to the covariates, we used the area under the receiver operator characteristics curves (AUROC) for analysis. We validated the frailty-scoring model for predicting outcomes by using bootstrap analysis (n = 10000). Values of *P <* 0.05 were considered statistically significant. All analyses and calculations were conducted using SPSS Statistics V21.0 (IBM Corporation, NY, USA) and STATA (STATA version 14.0, StataCorp LP, TX, USA).

## Results

### Study population

For a period of 1 year (2013), there were 7731 hospitalized elderly patients (aged ≥ 65 years) who were first admitted to the hospital and checked Cr levels during admission. Among them, only 632 patients underwent CGA within 1 year before the index hospitalization. We excluded 99 patients who were previously diagnosed with end-stage renal disease or had a baseline value of eGFR < 60 ml/min/1.73 m^2^. Consequently, 533 patients were included for this study (Fig 1). Participants’ mean age and eGFR were 76.3 years and 89.4 ml/min/1.73 m^2^, respectively. The median duration of hospital stay and mean duration of total follow-up were 12.7 days and 20.7 months, respectively. Of the 533, 47.3% were men, 29.1% had diabetes, 19.5% had hypertension, 3.6% had cardiovascular disease, and 59.8% had malignancy. In total, 297 patients (55.7%) underwent operation and 73 patients (13.7%) were admitted to the intensive care unit (ICU) during admission (data not shown).

### Frailty

Of total CGA variables, we chose five variables (ADL and IADL dependence, dementia, nutrition, and polypharmacy) because data on all these variables were completely available and there was no multicollinearity among the explanatory variables. Three CGA items (ADL dependence, dementia, and malnutrition), which had well-established cutoffs for severity, were scored (2, 1, or 0), and 2 items (IADL dependence and polypharmacy) were scored (1 or 0) by their reference values. The aggregate frailty score was calculated as sum of the component scores (range 1–8), and aggregate frailty scores were stratified per tertiles (Group 1: 1–2, Group 2: 3–4, Group 3: 5–8) (Fig 1).

A higher proportion of women and a lower proportion of malignancy and operation were observed in Group 3 compared with other groups. Older age; higher white blood cell count (WBC) and baseline eGFR; and lower body mass index, hemoglobin, albumin, and sodium were observed in the frailest group (Group 3) compared with other groups (Table 1).

**Table 1:** Baseline characteristics of patients according to frailty score.

	Group1Score: 1-2N = 183	Group2Score: 3-4N = 199	Group3Score: 5-8N = 151	P-value
**Demographics**				
Male (%)	116 (63.4)	83 (41.7)	53 (35.1)	**<0.001**
Age, years	73.8 ± 4.7	76.5 ± 5.4	79.0 ± 6.2	**<0.001**
Weight, kg	62.1 ± 9.4	55.7 ± 9.4	52.5 ± 10.1	**<0.001**
Body mass index	23.9 ± 3.0	23.0 ± 3.9	20.8 ± 3.4	**<0.001**
**Comorbidity**				
Diabetes mellitus (%)	43 (23.5)	67 (33.7)	45 (29.8)	0.089
Hypertension (%)	35 (19.1)	39 (19.6)	30 (19.9)	0.985
Cardiovascular disease (%)	7 (3.8)	7 (3.5)	5 (3.3)	0.968
Cerebrovascular disease (%)	5 (2.3)	6 (2.6)	10 (5.1)	0.593
Malignancy (%)	123 (67.2)	125 (62.8)	71 (47.0)	**0.001**
Systolic pressure (mmHg)	128.7 ± 17.6	129.6 ± 19.4	128.6 ± 22.7	0.859
Diastolic pressure (mmHg)	71.2 ± 11.9	72.0 ± 11.7	71.8 ± 13.1	0.790
**Laboratory values**				
Creatinine (μmol/L)	72.4 ± 14.5	66.9 ± 15.4	64.2 ± 16.3	**<0.001**
eGFR (ml/min/1.73m^2^)*	85.8 ± 20.8	89.1 ± 26.3	94.2 ± 40.9	**0.037**
WBC (10^9^/L)	7.54 ± 3.16	8.39 ± 6.83	9.43 ± 5.20	**0.006**
Hemoglobin (g/L)	124.0 ± 19.2	116.1 ± 19.7	112.3 ± 18.5	**<0.001**
Albumin (g/L)	37.6 ± 5.7	36.2 ± 5.5	34.9 ± 5.8	**<0.001**
Ca (mmol/L)	2.12 ± 0.17	2.11 ± 0.16	2.12 ± 0.18	0.947
P (mmol/L)	1.01 ± 0.22	1.03 ± 0.24	1.07 ± 0.28	0.128
Na (mmol/L)	138.5 ± 3.20	138.0 ± 3.6	136.5 ±5.3	**<0.001**
Total cholesterol (mmol/L)	4.22 ± 1.23	4.12 ± 1.33	4.12 ± 1.22	0.694
**Length of stay in hospital (days)**	12.0 ± 14.0	11.9 ± 13.5	14.0 ± 11.6	0.153
**Operation**	128 (69.9)	104 (52.3)	65 (43.0)	**<0.001**
**ICU admission**	20 (10.9)	28 (14.1)	25 (16.6)	0.324
**Discharge diagnosis**				
Benign tumor	5 (2.7)	6 (3.0)	1 (0.7)	
Cancer	112 (61.2)	112 (56.3)	58 (38.4)	
Cardiovasculocerebral disease	11 (6.0)	7 (3.5)	9 (6.0)	
Electrolyte imbalance	0 (0)	3 (1.5)	4 (2.6)	
Infection	30 (16.4)	29 (14.6)	31 (20.5)	
Musculoskeletal disease	11 (6.0)	22 (11.1)	31 (20.5)	
Non-infection GI disease	11 (6.0)	11 (5.5)	8 (5.3)	
Neuropsychiatric disease	2 (1.1)	7 (3.5)	5 (3.3)	
Weight loss	0 (0)	0 (0)	2 (1.3)	
Interstitial lung disease aggravation	1 (0.5)	0 (0)	2 (1.3)	
Uncontrolled diabetes mellitus	0 (0)	2 (1.0)	0 (0)	

Abbreviation: eGFR, estimated glomerular filtration rate; WBC, white blood cell count; ICU, intensive care unit; GI, gastrointestinal

*eGFR was estimated using the Modification of Diet in renal Disease (MDRD) study equation adjusted for Korean ethnic coefficient

### Development of AKI and AKI severity

In total, 54 patients (10.1%) developed AKI, and the median duration of AKI was 4.0 days (interquartile range: 1.0–11.0 days) after hospital admission. In terms of severity and dependency on renal replacement therapy, no difference was observed among the 3 groups (AKI stage 2–3: 1.6%, 4.5%, and 5.3%; renal replacement therapy dependency: 0.5%, 0.5%, and 0% for Groups 1, 2, and 3, respectively). In AKI group, a higher proportion of patients had hypertension, and ICU admissions were higher as compared to group without AKI. Additionally, AKI group showed higher eGFR and lower hemoglobin, albumin, and sodium levels than non-AKI group. The CGA revealed that patients with AKI were likely to have a higher ADL dependence and a poor cognitive function and nutritional condition. Further, more patients in AKI group had polypharmacy but this difference was insignificant (Table 2).

**Table 2:** Baseline characteristics of patients according to development of AKI.

	No AKIN = 479	AKIN = 54	P-value
**Demographics**			
Male (%)	224 (46.8)	28 (51.9)	0.566
Age, years	76.1 ± 5.7	77.7 ± 6.2	0.057
Weight, kg	56.7 ± 9.9	54.1 ± 12.3	0.147
Body mass index	22.8 ± 3.7	21.8 ± 3.8	0.081
Diabetes mellitus (%)	134 (28.0)	21 (38.9)	0.113
Hypertension (%)	87 (18.2)	17 (31.5)	**0.028**
Cardiovascular disease (%)	16 (3.3)	3 (5.6)	0.427
Cerebrovascular disease (%)	16 (3.3)	2 (3.7)	0.702
Malignancy (%)	288 (60.1)	31 (57.4)	0.770
Systolic pressure (mmHg)	129.0 ± 19.5	129.3 ± 21.8	0.920
Diastolic pressure (mmHg)	71.6 ± 12.2	72.3 ± 12.5	0.709
**Laboratory values**			
Creatinine (μmol/L)	68.8 ± 14.8	61.7 ± 21.2	**0.019**
eGFR (ml/min/1.73m^2^)*	86.7 ± 21.9	113.6 ± 62.5	**0.007**
WBC (10^9^/L)	8.2 ± 4.7	10.0 ± 9.3	0.168
Hemoglobin (g/L)	118.5 ± 19.8	110.8 ± 17.9	**0.007**
Albumin (g/L)	36.5 ± 5.6	34.4 ± 6.1	**0.011**
Ca (mmol/L)	2.12 ± 0.16	2.09 ± 0.20	0.264
P (mmol/L)	1.03 ± 0.22	1.08 ± 0.38	0.327
Na (mmol/L)	137.8 ± 3.6	135.7 ± 6.1	**0.015**
Total cholesterol (mmol/L)	4.19 ± 1.26	3.87 ± 1.26	0.075
**Comprehensive Geriatric Assessment**			
Polypharmacy, No (%)	243 (50.7)	33 (61.1)	0.154
Dependence, No (%)			
ADLs (partial and full)	154 (32.2)	28 (51.9)	**0.006**
IADLs	469 (97.9)	51 (64.4)	0.136
MMSE-KC score	21.6 ± 6.4	19.7 ± 6.7	**0.044**
SGDS-K-score	4.5 ± 3.7	5.6 ± 4.1	**0.038**
MNA score	22.0 ± 4.9	18.9 ± 5.8	**<0.001**
Mid-arm circumference, cm	23.2 ± 2.9	22.0 ± 2.8	**0.005**
**Length of stay in hospital (days)**	11.7 ± 10.6	21.6 ± 27.3	**0.011**
**Operation**	272 (56.8)	25 (46.3)	0.151
**ICU admission**	58 (12.1)	15 (27.8)	**0.003**

Abbreviation: eGFR, estimated glomerular filtration rate; WBC, white blood cell count; ADLs, activities of daily living; IADLS, activities of instrumental daily living; MMSE-KC, Korean version of the Mini-Mental State Examination; SGDS-K, Korean Geriatric Depression Scale; MNA, Mini Nutritional Assessment

*eGFR was estimated using the Modification of Diet in renal Disease (MDRD) study equation adjusted for Korean ethnic coefficient

**Fig 2:**
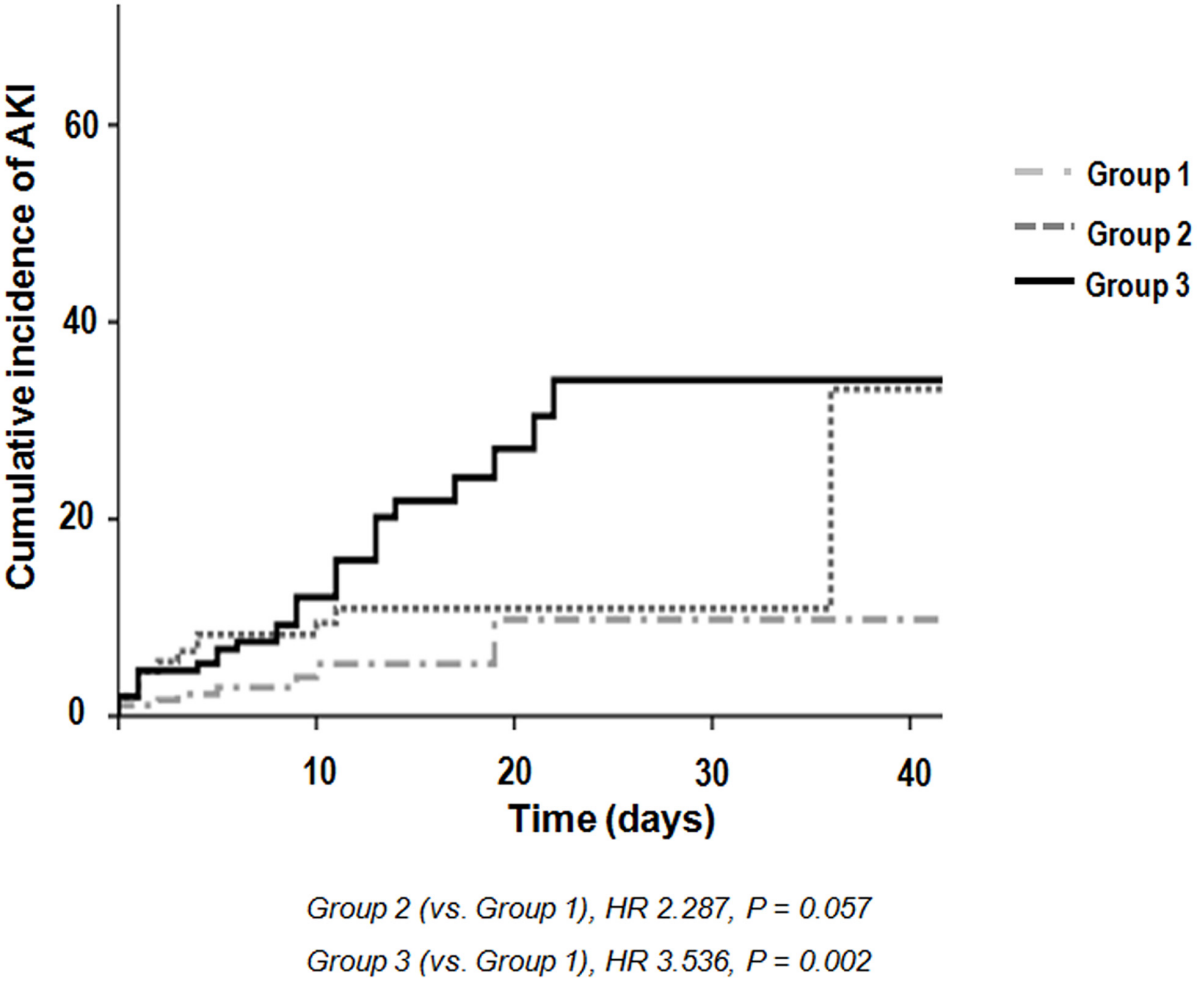
Cumulative incidence of acute kidney injury according to tertile of frailty score. AKI, acute kidney injury.

**Fig 3:**
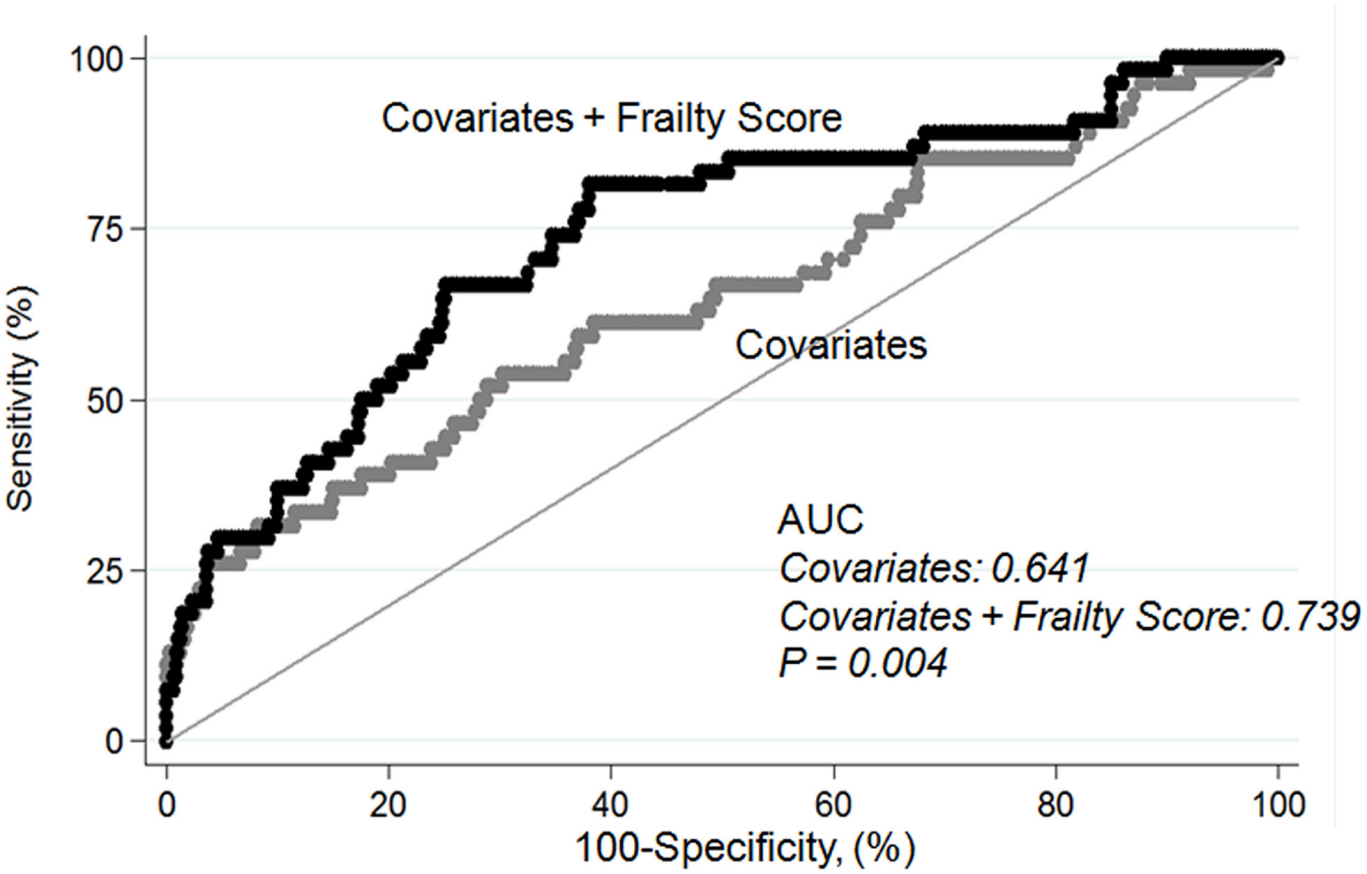
Area under receiver operating characteristics curve for development of acute kidney injury. Covariates including age, gender, BMI, cardiovascular disease, cerebrovascular disease, cancer, hypertension, diabetes, operation, intensive care unit admission, baseline creatinine, white blood cell count, hemoglobin, albumin, and sodium.

Evaluation of the relationship between tertiles of aggregate frailty score and development of AKI using multivariable Cox regression showed that the frailest group had an increased risk of AKI than other groups (reference: Group 1, HR = 3.536, *P* = 0.002) (Table 3 and Fig 2). The bias-corrected confidence interval was as follows by using bootstrap analysis (n = 10000) to validate the frailty score model for predicting outcomes: tertiles of aggregate frailty score (Group 3 vs Group 1) on AKI development, 1.607–10.486; on discharge to nursing facility, 1.504–6.328; on 90-day mortality, 2.354–33.119; and on 1-year mortality, 1.345–6.718. In patients with cancer, the effects of frailty on AKI were particularly apparent (reference Group 1, Group 3: HR = 7.829, *P* = 0.003). We found that discriminatory accuracy for AKI incidence substantially improved with the addition of tertiles of aggregate frailty score to covariates (AUROC 0.641, AUROC 0.739, *P* = 0.004) (Fig 3).

**Table 3:** Adjusted hazard ratios for association between frailty scores and development of AKI.

	Adjusted hazard ratio (95% CI)				
	N outcome/total	Multivariable^1^HR	P-value	Multivariable^2^HR	P-value
**Categorical variable**					
**Group 1**	**8/183**	Reference		Reference	
**Group 2**	**19/199**	2.542 (1.104–5.852)	**0.028**	2.287 (0.977–5.353)	0.057
**Group 3**	**27/151**	4.093 (1.835–9.132)	**0.001**	3.536 (1.571–7.958)	**0.002**

Abbreviation: AKI, acute kidney injury; HR, hazard ratio. Multivariable^1^, adjusted for age and gender; Multivariable^2^, adjusted for age, gender, BMI, cardiovascular disease, cerebrovascular disease, cancer, hypertension, diabetes, operation, intensive care unit admission, baseline creatinine, white blood cell count, hemoglobin, albumin, and sodium

### Discharge facility

**Fig 4:**
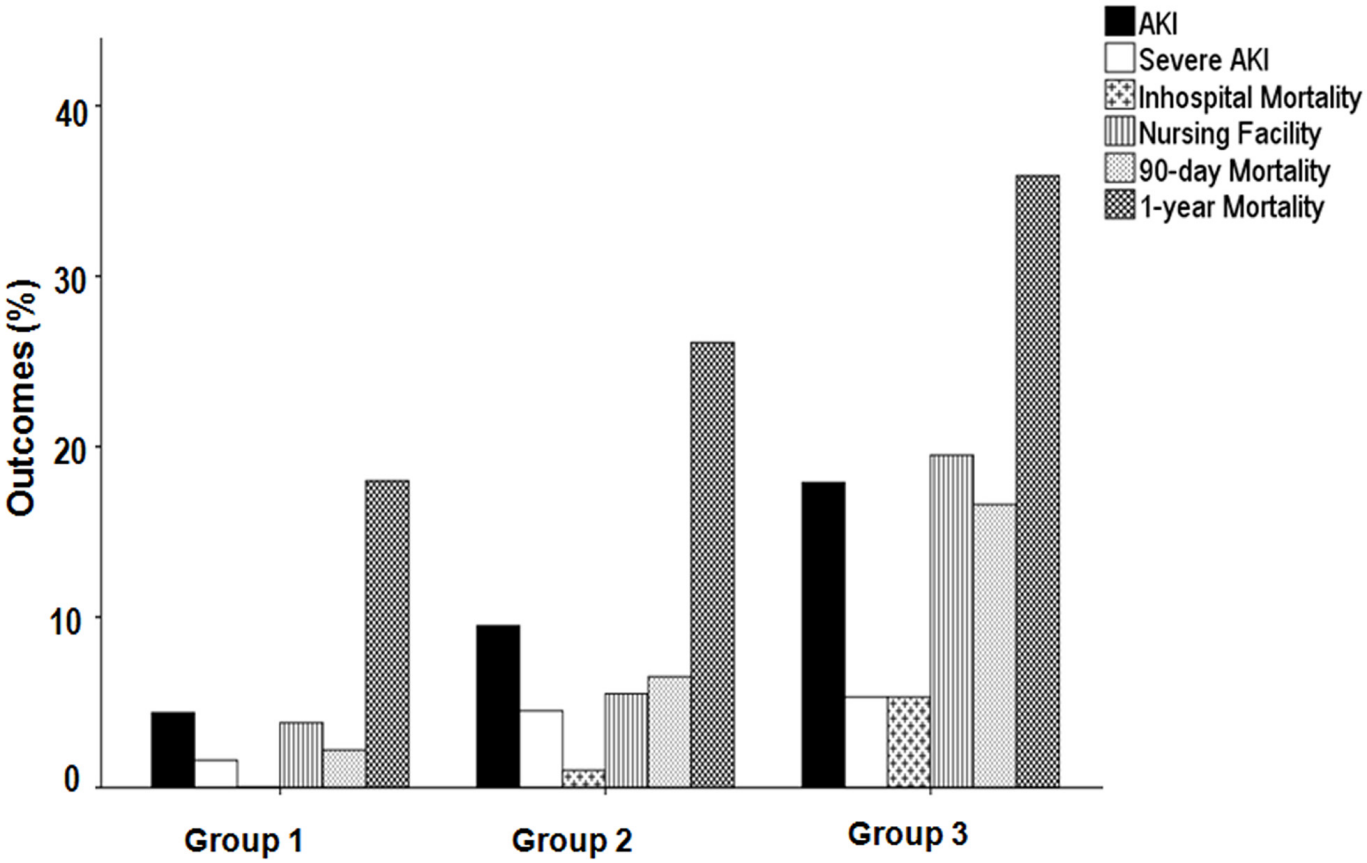
Incidence of outcomes according to tertile of frailty score.

Forty-six patients (8.6%) were transferred to nursing facilities, 477 patients (89.5%) were discharged home, and 10 patients (1.9%) died during admission. The associations between tertiles of aggregate frailty score and outcomes were revealed by multiple logistic regression analysis including baseline Cr and peak Cr levels during admission. The frailest group demonstrated an increased risk of discharge to nursing facility as compared to Group 1 (OR = 4.843, *P* = 0.002), whereas there was no significant difference between Groups 1 and 2 (Table 4 and Fig 4).

**Table 4:** Adjusted hazard ratios for association between frailty scores and outcomes.

	Adjusted hazard ratio (95% CI)				
	N outcome/total	Multivariable^1^HR	P-value	Multivariable^2^HR	P-value
**Discharge to nursing facility**	**N = 523**				
**Continuous variable, score**		1.611 (1.359–1.908)	**<0.001**	1.551 (1.286–1.870)	**<0.001**
**Categorical variable**					
Group 1	7/183	Reference		Reference	
Group 2	11/197	1.487 (0.564–3.922)	0.423	1.256 (0.417–3.782)	0.685
Group 3	28/143	6.122 (2.588–14.480)	**<0.001**	4.843 (1.800–13.032)	**0.002**
**90 day mortality**	**N = 533**				
**Continuous variable, score**		1.601 (1.340–1.914)	**<0.001**	1.498 (1.195–1.878)	**<0.001**
**Categorical variable**					
Group 1	4/183	Reference		Reference	
Group 2	13/199	3.728 (1.181–11.770)	**0.025**	1.640 (0.470–5.723)	0.438
Group 3	25/151	11.413 (3.787–34.394)	**<0.001**	6.555 (2.009–21.382)	**0.002**
**1-year all-cause mortality**	**N = 533**				
**Continuous variable, score**		1.286 (1.151–1.438)	**<0.001**	1.251 (1.076–1.455)	**0.004**
**Categorical variable**					
Group 1	33/183	Reference		Reference	
Group 2	52/199	2.039 (1.222–3.402)	**0.006**	1.284 (0.698–2.361)	0.422
Group 3	54/151	3.541 (2.069–6.057)	**<0.001**	3.249 (1.634–6.461)	**0.001**

Abbreviation: HR, hazard ratio. Multivariable^1^, adjusted for age and gender; Multivariable^2^, adjusted for age, gender, BMI, cardiovascular disease, cancer, hypertension, diabetes, operation, intensive care unit admission, systolic blood pressure, baseline creatinine, peak creatinine, white blood cell count, hemoglobin, albumin, sodium, and total cholestero

### Short-term (90-day) and long-term (1-year) ACM

The overall 90-day and 1-year ACM values for elderly inpatients were 7.9% and 26.3%(including 10 in-hospital deaths after admission), respectively. The short-term and long-term ACM rates were 2.2% and 18.0% in Group 1, 6.5% and 26.1% in Group 2, and 16.6% and 35.9% in Group 3 (*P*< 0.001 and *P* = 0.001). The frailest group demonstrated an increased risk of 90-day and 1-year ACM as compared to Group 1 (HR = 6.555, *P* = 0.002 and HR = 3.249, *P* = 0.001), but there were no significant differences between Groups 1 and 2 (Table 4 and Fig 4) in fully adjusted model including baseline Cr and peak Cr levels.

## Discussion

The main finding of this study was that frailty was a predictor for the development of AKI in elderly inpatients. Clinical outcomes, including the likelihood of discharge to nursing facility and short-term and long-term ACM, were associated with frailty, independent of the severity of AKI.

Old age is considered an independent risk factor for AKI[4]. Elderly patients with AKI have worse renal recovery rate and a higher mortality rate than younger patients[5, 6]. Frailty is a state of decreased physiological reserves, and frailty has been known to predict adverse outcomes in geriatric population[7–11]. However, the effect of frailty on the development of AKI in hospitalized elderly patients is unknown. This is the first study to investigate frailty as an independent risk factor for the development of AKI in elderly inpatients. Our findings may provide important information for treatment of geriatric inpatients.

On assessing hospitalized patients who underwent CGA before AKI, we found that frailty was associated with an increased risk of AKI in geriatric inpatients. The effect of frailty on the development of AKI can be explained as follows. First, frail patients and patients with AKI had similar characteristics including high WBC and low body mass index, hemoglobin, and albumin levels (Tables 1 and 2). Previous studies reported that a high WBC/body mass index is associated with AKI in critically ill patients[23, 24]. Hypoalbuminemia and anemia were also reported to be independently associated with AKI[25, 26]. Second, among the CGA variables that we chose, polypharmacy was associated with an increased risk of AKI in the elderly[4]. Third, frailty has been associated with several inflammatory cytokines including interleukin 6 and tumor necrosis factor-α [9, 27–29]. Overall, renal damage in AKI also involves inflammatory responses including immune cells, cytokines (i.e., interleukin 6 and tumor necrosis factor-α), and chemokines[30]. Therefore, frail patients might be vulnerable to AKI through the same inflammatory response. Fourth, frail patients have decreased physiological renal reserve: Hilmer reported that gentamycin clearance is significantly lower in frail patients compared to non-frail elderly patients after gentamycin infusion[31]. Furthermore, in our analysis, the addition of tertiles of aggregate frailty score to covariates improved discriminatory accuracy for predicting AKI. Therefore, frailty directly affects AKI incidence and is an independent risk factor for the development of AKI.

Our current finding that frailty is associated with an increased risk of short-term and long-term ACM and likelihood of discharge to nursing facility in elderly inpatients is consistent with that of recent studies[11, 32–35]. By using multivariable analysis after adjustment for baseline and peak Cr levels, we found that AKI affects associations between frailty and adverse outcomes, independent of the severity of AKI. Serum Cr levels may not always reflect renal dysfunction but also functional limitation in elderly people. However, there is a J-shaped association between Cr level and functional limitation in the elderly, which suggests that a lower serum Cr level may also be associated with functional limitation, muscle mass, and renal function[36]. Therefore, serum Cr level is not enough for predicting the development of AKI or clinical outcomes in elderly. The functional status in elderly patients may be one of the potential complementary predictors of AKI and other clinical outcomes in addition to serum Cr levels.

The frailty score comprised 5 categorical variables (ADL and IADL dependence, dementia, malnutrition, and polypharmacy) of the CGA. We chose these variables because data on all items were available and multicollinearity among the explanatory variables was excluded after analysis of variance inflation factor by logistic regression. The abovementioned variables might have different significances. Furthermore, each item had well-established cutoffs for severity and was scored according to the cutoffs. Internal validation was used by bootstrap analysis and prediction models were robust.

Our study has several limitations. First, this is a single-center study, and several participants with cancer were included in our analysis. Therefore, the results of our findings cannot be generalized. Second, we could not explain the exact cause of death that was associated with frailty and/or AKI because we did not have sufficient data. Third, with regard to defining AKI, we did not consider time (within 48 h or 7 days) according to the guideline proposed by KDIGO[22], but the majority of AKI episodes occurred in early period of admission (within 7 days) [37].

In conclusion, this study showed that frailty may independently predict the development of AKI in geriatric inpatients. Clinical outcomes, including the likelihood of discharge to a nursing facility and short-term and long-term mortality were associated with frailty, independent of severity of AKI. Further studies are needed to elucidate the pathophysiologic mechanism associating frailty and adverse outcomes.
